# Levels of Soluble E-Cadherin in Breast, Gastric, and Colorectal Cancers

**DOI:** 10.1155/2014/408047

**Published:** 2014-09-16

**Authors:** Ombretta Repetto, Paolo De Paoli, Valli De Re, Vincenzo Canzonieri, Renato Cannizzaro

**Affiliations:** ^1^Facility of Bio-Proteomics, Centro di Riferimento Oncologico (CRO), Aviano National Cancer Institute, Via F. Gallini 2, 33081 Aviano, Italy; ^2^Centro di Riferimento Oncologico (CRO), Aviano National Cancer Institute, Via F. Gallini 2, 33081 Aviano, Italy; ^3^Pathology Unit, Centro di Riferimento Oncologico (CRO), Aviano National Cancer Institute, Via F. Gallini 2, 33081 Aviano, Italy; ^4^Gastroenterology Unit, Centro di Riferimento Oncologico (CRO), Aviano National Cancer Institute, Via F. Gallini 2, 33081 Aviano, Italy

## Abstract

Soluble E-cadherin is a 80 kDa protein fragment coming from the proteolytic cleavage of the extracellular domain of the full length epithelial cadherin, a molecule involved in cell adhesion/polarity and tissue morphogenesis. In comparison with normal epithelia, cancer cells show a decreased cadherin-mediated intercellular adhesion, and sE-cad levels normally increase in body fluids (blood and urine). This review focuses on soluble E-cadherin in sera of patients affected by three solid cancers (breast, gastric, and colorectal cancers) and how its levels correlate or not with some cancer parameters (e.g., dimension, progression, and localisation). We will describe the main proteomics approaches adopted to measure sE-cad both *in vivo* and *in vitro* and the most important findings about its behaviour in cancer dynamics.

## 1. The Soluble E-Cadherin

The E-cadherins (E-cad), or “classical” cadherins of type I, belong to the large family of cadherins, transmembrane or membrane-associated glycoproteins, mediating cell-cell adhesion and playing a pivotal role in epithelial cell behaviour and tissue morphogenesis/remodelling (reviewed in [1–7]). Transcriptional E-cad reprogramming in epithelial cells leads to decreased adhesion and enhanced migration/invasion at the epithelial-to-mesenchymal transition (EMT) during cancer progression [8]. In this context, many cancer researches focus on E-cad expression and its modulation: basic structure of E-cadherin protein, posttranslational processing and maturation, genetic variants, gene expression (activation versus silencing), and transcript content/localization have been widely investigated, together with E-cad interactions with multiprotein complexes and signalling variations associated with alterations of E-cad cell-cell adhesion properties [9–12]. Most epithelial tumors loose E-cad partially or completely through mutation, epigenetic silencing, or increased expression of nonepithelial cadherins (colorectal CRC [13]; gastric cancer (GC) [14–16]; breast [17–22]; and GC and breast cancer [23]), and E-cad downregulation globally correlates with tumor grade and invasion. However, in human breast cancer, Hollestelle et al. [24] recently have observed that E-cad loss was neither causal nor necessary for EMT. At protein level, under pathological conditions, the effects of E-cad-associated genetic changes are usually evaluated in terms of content and localization by* in situ* hybridization and immunostaining (e.g., [14, 15]).

Other mechanisms potentially influencing E-cad normal functions such as its binding to other proteins include the levels of its phosphorylation together with specific proteolytic events [4]. Indeed, enzymes such as secretases, calpain, and caspases may cleave E-cad in its cytoplasmic part, while matrix metalloproteinases (e.g., MMP-2, MMP-3, MMP-7, MMP-9, and MMP-14, stromelysin-1, and matrilysin) and cathepsins (B, L, S), together with other proteases (e.g., disintegrins AAM10 and AAM15 and plasmin), secretases, calpain, and caspases, besides bacterial proteases, can cleave E-cad ectodomain near the plasma membrane and generate a soluble 80 kDa E-cad fragment (sE-cad) released in the extracellular space [4, 25].

At present, serum levels of sE-cad are known to increase in patients affected by cancer (e.g., breast, gastric, and colorectal cancers; Table 1) in respect to healthy patients, so that there is a growing interest in sE-cad as “candidate sentinel molecule” in cancer research (reviewed by [25–27]). In these cases, the sE-cad levels have been associated with metastatic disease and worse prognosis, and the E-cad cleavage into sE-cad has been linked to malignant adenoma-cancer progression. However, sE-cad may be also increased due to oxidative stress [28] and production of cytokines involved in inflammation and tumorigenesis [25, 29].

Generally, since the first observations in 1990, the global decrease in E-cad in dissociating/metastasising cancer cells was accompanied by an increase in sE-cad fragments in patient sera, so that the first emerging idea was to consider the soluble sE-cad as originating from the rapid turnover of tumor cells and to relate the sE-cad concentration to the tumor size.

Here, we report proteomics applied to the characterization of sE-cad amount in three solid cancers (breast, gastric, and colorectal cancers) and describe the most common techniques adopted since sE-cad discovery. Since sE-cad presence is not only limited to these three pathologies, we also briefly summarized the findings of other works in a recapitulative table (Table 1).

## 2. Proteomics Approaches Applied to Cadherin Characterization 

### 2.1. Immunoenzymometric Assay

The protocol by Katayama et al. [27]—modified after Katayama et al. [30]—has been widely adopted to measure serum sE-cad concentrations with a commercially available sandwich ELISA kit. Briefly, as it has been described in Katayama et al. [30], the first monoclonal antibody (the human E-cadherin-1 monoclonal antibody, HECD-1, raised against the cad extracellular domain) is coated onto microtiter plate wells and creates the solid phase. Nonspecific binding is blocked by a buffer. Serum samples from patients and standard solutions are then incubated in the microtiter plate wells. The second monoclonal antibody, SHE 13-1, labelled with peroxidase is added. During incubation, human E-cad molecule is trapped by the two monoclonal antibodies as a sandwich, so that the technique is also reported as sandwich-type immunoenzymometric assay (IEMA). The reaction between the peroxidase and substrate solution results in colour development with intensities proportional to the concentration of human sE-cad in serum samples and standards. The absorbance of the developed colour is measured at 450 nm. Accurate sample concentrations of human E-cad are determined by comparing specific absorbance with those obtained from the standards plotted on a standard curve. The authors advised the use of serum for routine in cadherin assay, since dissolved Ca^2+^ is not artificially removed by chelation and a coexistent Ca^2+^ is necessary to stabilize the structure of sE-cad immunoreactive with those monoclonal antibodies.

### 2.2. Western Blotting (WB)

In most reports, in patients, the sE-cad amount is also evaluated with WB after protein separation by one-dimensional acrylamide gel electrophoresis (1-DE), and it can be compared with the full length E-cad expression, which in turn is analysed by immunostaining* in situ. *WB analyses reveal the presence of multiple bands, among which are the full length E-cad at 120 kDa and the sE-cad at 80 kDa.

### 2.3. Reverse Phase Protein Array (RPPA)

Another targeted approach was used by Perez-Rivas et al. [31] to analyse the expression levels of 42 selected serum soluble proteins using the reverse phase protein array technology (RPPA) (described by [32]). The RPPA represents a high-throughput and sensitive technology platform for quantitative and multiplexed immunoassays, in which small amounts of cell lysates or fluids (e.g., serum and urine) are immobilized on individual spots onto a capturing surface called microarray that is then incubated with a single specific antibody to detect the expression of the target protein across many samples. Detection is performed using either a primary or a secondary labeled antibody by chemiluminescent, fluorescent, or colorimetric assays, the array is then imaged, and the obtained data are quantified.

## 3. Soluble E-Cadherin in Breast Cancer 

In BC patients, first studies started in 2005 when Hofmann and colleagues measured sE-cad levels in sera of 133 patients before and after neoadjuvant chemotherapy with an enzyme-based immunoassay technique, and they positively correlated them with the pre- and posttherapeutic tumor size as well as the disease-free interval [33]. In the same year, Dollé et al. [34] evaluated by 1-DE and WB (anti-HECD-1) the release of sE-cad in the media of MCF-7/AZ BC cells grown in presence or absence of the nerve growth factor (NGF), a small secreted protein that is important for the development and survival of certain target neurons, and their results supported a relation between sE-cad levels and the BC cell acquisition of an invasive phenotype. In order to identify markers for BC patient response to surgery, the following analyses were addressed to characterize the differential serum proteomes “before versus after surgery” by RPPA [31]. Among the 45 polyclonal antibodies, sE-cad resulted to be increased after surgery in sera of invasive tumor patients as compared with those of healthy women and patients with noninvasive tumors. The potential value of sE-cad as marker to predict response(s) to or to assess prognosis after preoperative systemic chemotherapy (PST) was further tested in sera of 108 patients with locally advanced BC using a commercially available ELISA kit [35]. Soluble E-cad level was significantly lower in sera of preoperatively treated BC patients undergoing PST with pathological complete response (pCR), suggesting a putative role of sE-cad as a predictive marker for BC patients for chemotherapy response. In the same year, Brouxhon et al. [36, 37] explored whether endogenous sE-cad levels (i) were overexpressed in HER2+ human and mouse BC specimens and tumors and cell culture systems of human triple-negative BC (TNBC), which does not express the genes for estrogen and progesterone receptors, and Her2/neu, and (ii) exerted prooncogenic effects via modulation of the HER-PI3K/Akt/mTOR-IAP axis and/or synergism with the HER ligand EGF. Immunoprecipitation assays were performed after cell lysate incubation with EGFR/HER1, HER2, HER3, HER4, or E-cad ectodomain-specific antibodies, and the immune complexes were then separated through acrylamide 1-DE, while sE-cad levels were quantified using human E-cadherin Quantikine ELISA Kits in sera, urines, and cell conditioned media. Globally, sE-cad protein expression levels increased in the all tested experimental systems, sE-cad contributed via HER family members to enhance MAPK, PI3K/Akt/mTOR, and IAP signaling, it played prooncogenic functions in both HER2+ and TNBC cell lines, and* in vitro* it acted together with the EGF ligand to promote BC proliferation, migration, and invasion.

## 4. Soluble E-Cadherin in Gastric Cancer 

In GC patients, sE-cad fragments were firstly investigated by Gofuku et al. [42] who compared the amount of serum sE-cad in 81 GC patients: sE-cad levels were measured [27], and they resulted to be significantly higher in GC patients than in healthy controls, with the highest sE-cad increase being revealed in the GC tissues having a partially reduced expression of the full length E-cad. In this work, sE-cad levels decreased after tumor removal by surgery. This same assay was further adopted by Chan et al. [41], who found significantly higher sE-cad concentrations in GC patients (*nr* = 116), correlating them with tumor size and carcinoembryonic antigen (CEA) amount. The same team further correlated serum levels of sE-cad with protein expression in a trial of 116 patients [39]: the sE-cad was found as an independent factor predicting long-term survival, with 90% of patients with a serum level of sE-cad > 10,000 ng/mL having a survival time < 3 years. In the same year, by comparing GC patients classified as intestinal-type (51%) to those with a diffuse-type (49%) according to Laurén, Juhasz et al. [40] showed that serum sE-cad levels increased in intestinal-type GC, especially in advanced stages, whereas in the diffuse-type sE-cad levels decreased in advanced and metastasized cases. The sE-cad concentrations were thus proposed to be interpreted along with Laurén classification and to represent a negative prognostic marker in the only intestinal GC type. Blood samples of 69 GC patients who underwent curative surgery were assayed prospectively (preoperatively, 1 month after surgery and every 3 months thereafter) for sE-cad and compared to CEA using the method described by Katayama et al. [27, 38]: this study documented the use of sE-cad levels in predicting and detecting recurrent GC in the first 3–6 months after patients undergo curative surgery, with a sensitivity significantly better compared to that one of CEA with the reported cut-off level. The importance of these findings to stratify patients into “low-” and “high-” risk groups for consideration of more aggressive adjuvant therapy and vigilant surveillance in the hope of improved survival was evidenced by the authors.

## 5. Soluble E-Cadherin in Colorectal Cancer 

Assays for the detection of sE-cad in blood samples of CRC patients were firstly performed by Velikova et al. [47] with the IEMA assay: in CRC patients versus healthy subjects, though elevated in some patients, sE-cad concentrations were not significantly elevated compared with those of the control group, which was different from what was observed for serum concentrations of intercellular adhesion molecule-1 (ICAM-1) and vascular cell adhesion molecule-1 (VCAM-1), which were significantly elevated. Potential marker characteristics for sE-cad in CRC were further evidenced by Wilmanns et al. [46], who however excluded a routine clinical use due to a lack of specificity. In particular, sE-cad concentrations were increased not only in CRC patients, but also in benign disease controls, and they correlated with the primary tumour or “T-stage” (UICC-TNM classification) and serum CEA in case of existing liver metastases. In 2007, concomitant to the development of protocols for the total proteins released by cells, also known as “secretome,” Diehl et al. [62] presented two-dimensional acrylamide gel electrophoresis (2-DE) maps for the secretome released by SW620 human CRC cells* in vitro*. Total proteins were compared with the secreted ones by differential in gel electrophoresis (DIGE) in order to search for the secretome-specific proteins of CRC cells. Among the identified secretome-specific spots, there was the sE-cad (accession number: gi|6682963; experimental versus theoretical pI and MW: 4.2/4.6 and >85/99.7). However, the prognostic value of sE-cad level has not been clearly demonstrated yet. In a further study from 186 CRC patients, serum sE-cad levels were related to clinicopathological findings [44]. Preoperative elevated sE-cad levels were found to be associated with poor prognosis in CRC and, in particular, sE-cad measures were thought to provide valuable information for predicting prognosis in patients with hepatic metastasis. Previously, Weiß et al. [45] compared sE-cad levels in sera from patients with CRC, colorectal adenoma, inflammatory bowel disease, and familial adenomatous polyposis, and they found a significant increase in sE-cad load in patients with late-stage CRC (Stages III and IV). In individual patients with late-stage CRC, sE-cad serum levels were proposed to directly reflect their disease status over time. These results suggested a potential application of sE-cad as an alternative diagnostic biomarker for monitoring disease particularly in patients with CAE negative tumors.

## 6. Conclusion

All the analyses hereby described the evidence that the serum sE-cad levels generally increase in many cases concomitant to the decrease in full length E-cad expression in tissues, sE-cad, thus behaving like a putative “disease sentinel.” Because of its stability in serum, sE-cad accumulating in the blood may serve as sensitive indicator of tumor-associated proteolysis and potential candidate for an early disease detection. However, when the sE-cad decrease in patient sera is associated with particular cancer characteristics (e.g., stage, size, and localization), therapy (e.g., before or after therapy; before or after surgery), or other clinical parameters (e.g., levels of markers such as CEA or other adhesion molecules), it becomes hard to generalize findings and extremely difficult to propose the sE-cad protein as prognostic marker in clinical trials. The sE-cad molecule like CEA does not appear to be adequate for screening purposes due to a lack of specificity and sensibility; however, they both could be tested in association with monitor therapies. Proteomics offers a powerful approach to go further with sE-cad characterization. For instance, it may be tempting to analyze sE-cad levels in parallel with those of other key molecules or markers and to characterize sE-cad associated with CDH1 mutations at gene level both* in vivo* and* in vitro* or to expand knowledge about the candidate molecules triggering cleavage of the full length E-cad into its soluble form. Moreover, the parallel characterization of the secretome will help to better decipher the surprisingly abundant subset of subproteome, which is partly constituted by ectodomains of membrane proteins cleaved from the cell surface such as sE-cad.

## Figures and Tables

**Table 1:** List of the major works reporting soluble E-cadherin (sE-cad) proteins in the last 20 years.

Tumor	Location	Major findings	References
	Serum and urine	sE-cad exerted prooncogenic effects	Brouxhon et al., 2013 [36]
	Serum	sE-cad may be a candidate marker for the prediction of response to PST	Hofmann et al., 2013 [35]
BC	Serum	Preoperative sE-cad levels were higher in invasive tumor and changed after surgery	Perez-Rivas et al., 2012 [31]
	Cell medium	sE-cad release appeared to be regulated by NGF during the BC cell induction of an invasive phenotype	Dollé et al., 2005 [34]
	Serum	sE-cad levels positively correlated with the pre- and posttherapeutic tumor size as well as the disease-free interval	Hofmann et al., 2005 [33]

	Serum	sE-cad was a good marker predicting the disease recurrence in the first 3–6 months after surgery	Chan et al., 2005 [38]
	Serum	sE-cad was an independent factor predicting long-term survival, and it was proposed as a valuable pretherapeutic prognostic factor in GC	Chan et al., 2003 [39]
GC	Serum	sE-cad levels increased in intestinal-type GC, especially in advanced stages, and sE-cad was proposed as prognostic marker	Juhasz et al., 2003 [40]
	Serum	sE-cad resulted in a potential valid prognostic marker for GC	Chan et al., 2001 [41]
	Serum	Tumor tissues showed higher sE-cad levels, which were proposed as excellent tumor markers with high sensitivity	Gofuku et al., 1998 [42]

	Secretome∗	More than 170 proteins were identified, including the E-cad	Imperlini et al., 2013 [43]
	Serum	An elevated sE-cad level was a risk factor for predicting poor prognosis and hepatic metastasis	Okugawa et al., 2012 [44]
CRC	Serum, secretome, and tissue	In patients with late-stage CRC, serum sE-cad levels directly increased and they were proposed as diagnostic biomarker	Weiß et al., 2011 [45]
	Serum	sE-cad was proposed as potential marker but not for a routine clinical use because of the lack of specificity	Wilmanns et al., 2004 [46]
	Serum	Even if elevated in some patients, sE-cad concentrations were not significantly elevated compared with those of controls	Velikova et al., 1998 [47]

BlC	Urine	Urinary sE-cad values were not greater than urinary total protein content	Protheroe et al., 1999 [48]
	Serum	sE-cad concentrations were higher in BlC patients, and they were correlated with known prognostic factors	Griffiths et al., 1996 [49]
BlC and urinary disorders	Urine	This is the first evidence of sE-cad forms in urine, which were proposed for diagnosis and prognosis of urinary disorders	Banks et al., 1995 [50]
TCC of the urinary BlC	Urine	sE-cad levels were higher in TCC patients and they strongly correlated with tumor grade	Shi et al., 2008 [51]
	Cell medium and urine	Higher sE-cad levels were associated with positive cytology results and muscle invasive tumor stage	Shariat et al., 2005 [52]
	Serum	Higher levels of sE-cad correlated with increasing tumor grade but not with clinicopathological stage	Durkan et al., 1999 [53]
MIBlC	Plasma	Preoperative plasma levels of sE-cad were higher in patients with metastases to regional and distant lymph nodes	Matsumoto et al., 2003 [54]

HCC	Serum	sE-cad levels were elevated in HCC patients and their levels were proposed as potential HCC prognostic marker	Soyama et al., 2008 [55]

HD	Serum	In HD patients, at diagnosis, sE-cad levels decreased, even if they were not statistically significant, in comparison to healthy controls	Syrigos et al., 2004a [56]

LC	Serum	Circulating sE-cad levels were higher in NSCL patients, but without any statistical evidence as prognostic factor of cell survival	Syrigos et al., 2004b [57]

MM	Serum	Circulating sE-cad levels were higher in MM patients and were proposed as prognostic factor of cell survival	Gogali et al., 2010 [58]
	Cell medium	sE-cad influenced tumor invasion and it was proposed to play multiple functional roles than cell adhesion	Charalabopoulos et al., 2006 [59]

PCa	Serum	sE-cad concentration highly statistically decreased after surgery in 82% patients	Iacopino et al., 2012 [60]
	Serum and tissue	This was the first report of sE-cad detection in neoplastic PCa, and sE-cad was proposed as biomarker for PCa disease progression	Kuefer et al., 2003 [61]

SCC	Serum	sEcad induced SCC proliferation and migration and was proposed as a therapeutic target in coetaneous SCCs	Brouxhon et al., 2014 [37]

BlC: bladder cancer; BC: breast cancer; CRC: colorectal cancer; GC: gastric cancer; HCC: hepatocellular carcinoma; HD: Hodgkin's disease; LC: lung cancer; MAPK: mitogen-activated protein kinase; MIBlC: muscle invasive bladder cancer; MM: multiple myeloma; NGF: nerve growth factor; PCa: prostate cancer; PST: preoperative systemic chemotherapy; SCC: skin squamous cell carcinoma; sE-cad: soluble E-cadherin; TTC: transitional cell carcinoma; ^*^secretome: serum-free cellular culture.

## Abbreviations

1-DE: One-dimensional acrylamide gel electrophoresis
2-DE: Two-dimensional acrylamide gel electrophoresis
BlC: Bladder cancer
BC: Breast cancer
CEA: Carcinoembryonic antigen
CRC: Colorectal cancer
DIGE: Difference gel electrophoresis
ELISA: Enzyme linked immunospecific assay
GC: Gastric cancer
HD: Hodgkin’s disease
HER: Human epidermal growth factor receptor
IEMA: Immunoenzymometric assay
IGF-1R: Insulin-like growth factor-1 receptor
MAPK: Mitogen-activated protein kinase
MIBlC: Muscle invasive bladder cancer
MM: Multiple myeloma
MMP: Matrix metalloproteinase
MS: Mass spectrometry
mTOR: Mammalian target of rapamycin
NCT: Neoadjuvant chemotherapy
NSCLC: Non-small cell lung cancer
PAGE: Polyacrylamide gel electrophoresis
PCa: Prostate cancer
PI3K: Phosphatidylinositol 3-kinase
PST: Preoperative systemic chemotherapy
SCC: Skin squamous cell carcinoma
SCLC: Small cell lung cancer
SDS: Sodium dodecyl sulphate
sE-cad: Soluble E-cadherin
sICAM-1: Soluble intercellular adhesion molecule-1
sVCAM-1: Soluble vascular cell adhesion molecule-1
TTC: Transitional cell carcinoma
TURBT: Transurethral resection of bladder tumor
WB: Western blotting.
